# Understanding Health Inequalities Through the Lens of Social Epigenetics

**DOI:** 10.1146/annurev-publhealth-052020-105613

**Published:** 2022-04-05

**Authors:** Chantel L. Martin, Lea Ghastine, Evans K. Lodge, Radhika Dhingra, Cavin K. Ward-Caviness

**Affiliations:** 1 Department of Epidemiology, Gillings School of Global Public Health, University of North Carolina at Chapel Hill, Chapel Hill, North Carolina, USA; 2 Carolina Population Center, University of North Carolina at Chapel Hill, Chapel Hill, North Carolina, USA; 3 School of Medicine, University of North Carolina at Chapel Hill, Chapel Hill, North Carolina, USA; 4 Department of Environmental Sciences and Engineering, Gillings School of Global Public Health, University of North Carolina at Chapel Hill, Chapel Hill, North Carolina, USA; 5 Institute of Environmental Health Solutions, University of North Carolina at Chapel Hill, Chapel Hill, North Carolina, USA; 6 Center for Public Health and Environmental Assessment, US Environmental Protection Agency, Chapel Hill, North Carolina, USA

**Keywords:** DNA methylation, health inequalities, psychosocial stressors, racism, socioeconomic disadvantage, epigenetic mechanisms

## Abstract

Longstanding racial/ethnic inequalities in morbidity and mortality persist in the United States. Although the determinants of health inequalities are complex, social and structural factors produced by inequitable and racialized systems are recognized as contributing sources. Social epigenetics is an emerging area of research that aims to uncover biological pathways through which social experiences affect health outcomes. A growing body of literature links adverse social exposures to epigenetic mechanisms, namely DNA methylation, offering a plausible pathway through which health inequalities may arise. This review provides an overview of social epigenetics and highlights existing literature linking social exposures—i.e., psychosocial stressors, racism, discrimination, socioeconomic position, and neighborhood social environment—to DNA methylation in humans.We conclude with a discussion of social epigenetics as a mechanistic link to health inequalities and provide suggestions for future social epigenetics research on health inequalities.

## 1. INTRODUCTION

Longstanding inequalities in morbidity and mortality along racial/ethnic lines persist in the United States. For example, Black children are three times more likely to be born prematurely and have lower birth weights than White children, and Black adults have higher rates of hypertension, several cancers, and shorter life expectancy than their White counterparts (4, 36, 45, 46, 83, 93). Although the determinants of health inequalities are multifaceted and complex, substantial evidence points to social and structural factors produced by inequitable and racialized systems as contributing sources (8, 9). Individuals from racially and ethnically minoritized groups (i.e., Black Americans, Latinx, and Indigenous people) are more likely than White individuals to reside in segregated neighborhoods and to experience social disadvantages and psychosocial stressors, all of which are associated with health inequalities (26, 53, 78, 94, 133). Less understood are the biological pathways through which adverse social exposures contribute to disparate health outcomes. Social epigenetics seeks to address this research gap by elucidating how social exposures reflecting systemic inequities get under the skin to influence health outcomes and produce health inequalities. This review offers an overview of social epigenetics and highlights existing research linking social exposures to epigenetic mechanisms in humans, with an emphasis on DNA methylation (DNAm). The review is written from a US-centric perspective on health inequalities, but it draws on literature from other countries. We conclude with a discussion of social epigenetics as a plausible mechanistic link from social exposures to health inequalities and provide suggestions for future social epigenetics research.

## 2. SOCIAL EPIGENETICS: A PROMISING FIELD FOR UNDERSTANDING MECHANISMS OF HEALTH INEQUALITIES

As defined by Krieger’s ecosocial theory, the concept of embodiment describes the process by which humans biologically incorporate the lived experiences of their environment (66, 67). Developmental origins of health and disease (DOHaD) and the weathering hypothesis offer two conceptual frameworks for investigating biological embodiment and population patterns of health (49). DOHaD posits that exposures, including social stressors, during early development—from gestation to early childhood—predispose individuals to a specific health trajectory (15, 69). The impact of stress on the epigenome is thought to persist across generations via three possible pathways: (*a*) transmission of epigenetic marks via germline cells, (*b*) maternal experiences of stress influencing fetal epigenetic programming,and (*c*) increased likelihood of experiencing social stressors in the offspring of parents who themselves experienced social stressors (29, 31). Alternatively, the weathering hypothesis postulates that racial health inequalities are a result of chronic exposure to adversity and marginalization that leads to earlier onset of physiological dysregulation and aging (48, 50). Emerging research proposes epigenetic mechanisms as pathways underlying both the DOHaD and weathering processes (49).

Social epigenetics is an emerging area of research aimed at identifying mechanisms of health inequalities at a molecular level (5, 25, 32, 107, 113, 122, 123). Epigenetic mechanisms respond to exogenous exposures and alter gene expression without changing the underlying genetic sequence (18). Epigenetic changes are increasingly accepted as markers and potential mediators of differential aging and life expectancy (54), and they may represent one mechanism by which deleterious social and economic exposures alter immune function, increase systemic inflammation, and influence other markers of complex chronic disease in the context of documented health inequalities (43, 128, 139). There are three types of epigenetic mechanisms: (*a*) microRNAs, (*b*) histone modifications, and (*c*) cytosine-phosphate-guanine (CpG) methylation (also referred to as DNAm). Though these three epigenetic mechanisms interact (13, 102), they are often studied independently, with DNAm as the most widely studied epigenetic mechanism and the focus of this review.

The primary form of DNAm studied is 5-methylcytosine—the addition of a methyl group to the fifth carbon of the cytosine at a CpG dinucleotide site. Other forms of DNAm are less studied due to assay complexity (e.g., hydroxymethylation) or to relative rarity (e.g., noncytosine methylation is generally present only during development in humans). The biomolecular mechanisms by which a methyl group is added to (methylated) or removed from (demethylated) a cytosine are detailed in another review by Martin & Fry (82). Although DNAm is not necessarily the most important epigenetic regulatory feature, high-throughput chip-based technologies have made it the most accessible for scientific inquiry across a broad range of disciplines. The ease and availability of this DNAm microarray technology has resulted in an explosion of research; therefore, the bulk of the epigenetic literature comprises studies relating DNAm to a wide variety of exposures and health outcomes.

At a given CpG site in a given cell, a methyl group is either present (methylated) or absent (unmethylated). When measuring DNAm in tissue samples, microarray technologies are used to estimate the proportion of cells for which a given CpG locus is methylated. Global methylation is commonly measured using repetitive DNA elements—i.e., LINE-1 and Alu methylation (59). High-throughput array technology has made possible measuring DNAm across hundreds of thousands of CpG sites. With the current array technology, methylation proportion can be estimated for more than 850,000 CpG sites in each DNA sample (see Illumina’s sequencing and array-based solutions for genetic research at https://www.illumina.com). Statistical approaches have been developed to analyze this large number of CpG sites in relation to variables of interest; yet limited sample sizes, computing resources, and analytic expertise have posed uphill challenges to analyzing epigenome-wide DNAm data. As a result, regional methylation and candidate gene analyses are commonly utilized.

Epigenetic clocks are a class of composite metrics that use the degree of methylation at dozens to thousands of CpG sites to estimate biological age (75). Whereas first-generation clocks (i.e., Hannum and Horvath clocks) were calibrated using only chronological age, later clocks (e.g., Levine’s PhenoAge) estimate biological age using chronological age along with indicators of phenotypic state or health outcome. Some clocks (e.g., Hannum, Horvath, and Levine) can be estimated using only DNAm data, which makes them a true readout of biological aging based on molecular measures. Others (e.g., GrimAge) incorporate chronological age and other surrogate measures into their estimations, which makes them better predictors of mortality risk at the cost of needing information beyond the methylation profiles of a sample (79). Accelerated epigenetic aging occurs when an individual’s estimated DNAm age is greater than their chronological age. For all developed clocks, a greater age acceleration is associated with increases in mortality risk and, in many cases, functional deficits, molecular changes, and chronic disease incidence (38, 54). Below, we highlight empirical studies examining associations of social exposures— psychosocial stressors, discrimination and racism, socioeconomic position (SEP), and neighborhood social environment—with DNAm patterns and epigenetic aging, with a particular focus on stress-response and inflammatory pathways.

## 3. PSYCHOSOCIAL STRESSORS AND DNA METHYLATION

Some of the earliest social epigenetics studies were on exposure to social adversities in relation to DNAm. In this section, we describe studies of the following psychosocial stressors: abuse, daily stressors, cumulative lifetime stress, financial stress, war-related stress, adverse childhood events, significant life events, and exposure to violence (summarized in Table 1).

### 3.1. Early Life Studies

Several studies using targeted approaches (i.e., candidate gene analyses) have shown that early life psychosocial stressors [i.e., prenatal exposure to intimate partner violence (IPV), war-related stress, and child abuse] are associated with modifications in DNAm at the *NR3C1* gene (100, 101, 114, 124). *NR3C1* encodes for the glucocorticoid receptor and regulates hypothalamus-pituitary-adrenal (HPA) axis functioning. HPA axis development begins during gestation and plays a mediating role in stress response (56). Exploratory findings suggest that associations between prenatal stressors and methylation at the *NR3C1* gene may be sex-specific, though additional research is needed to confirm these results (100). A body of research using data from a mother-newborn cohort in the Democratic Republic of the Congo explored prenatal stress and DNAm patterns in other stress-related genes, in addition to *NR3C1*, finding that maternal exposure to chronic stress, war-related stress, and sexual assault was associated with increased DNAm at sites located in the *NR3C1*, *BDNF*, *CRH*, *CRHBP*, *FKBPF*, and *IGF1* genes (61, 62, 90, 91).

There is also evidence suggesting that DNAm marks associated with exposure to childhood adversity may be observable into adulthood. A study among Black women identified differential methylation at the *NR3C1* gene among those women who had experienced abuse as children, with the associations increasing with severity of abuse (114). Interestingly, childhood emotional support modified the effects among women reporting the highest levels of physical and sexual abuse during childhood. These findings are consistent with a study of young Black men, which found that prosocial ties with parents, peers, partners, and mentors were inversely associated with DNAm at *OXTR*, a stress-related gene that encodes the oxytocin receptor (64). Taken together, these findings suggest that positive aspects of the social environment (i.e., social support) may buffer effects of social adversity on DNAm. Additionally, differential methylation at serotonin transporter genes, *SLC6A4* and *5HTT,* has also been investigated in relation to childhood abuse and maltreatment in two separate studies, which found associations between childhood abuse and *SLC6A4* and *5HTT* methylation in promoter regions in adulthood (16, 17).

Associations between childhood adversity and epigenome-wide DNAm have also been examined using array-based technologies (39, 41, 55, 60, 84, 105). Essex et al. (41) identified developmental window- and sex-specific DNAm patterns in response to maternal and paternal stress exposure. Specifically, maternal stress during infancy was associated with increased methylation at 139 CpG sites, while paternal stress was associated with increased methylation at 31 CpG sites during preschool years. Additional sex-specific analyses revealed stronger associations between paternal stress and differential methylation in girls,whereas maternal stress showed stronger associations with differential methylation in boys. Additional support for the idea that developmental timing matters was provided by a more recent study by Dunn et al. (39). The authors examined the associations between seven adversities (caregiver physical or emotional abuse, sexual or physical abuse, maternal psychopathology, one adult in the household, family instability, financial stress, and neighborhood disadvantage) during three life stages: very early childhood (before 3 years of age), early childhood (3–5 years of age), and middle childhood (6–7 years of age) on epigenome-wide DNAm. The authors identified 38 CpG sites associated with adversities. Of the 38 CpG sites identified, 22 CpG sites were differentially methylated in response to exposure during the very early childhood period.

Differential methylation in relation to child abuse has also been examined in DNA from sperm cells. Roberts et al. (105) identified 12 differentially methylated regions (DMRs) in sperm DNA among men who experienced abuse in childhood, including genes related to immune function (*SDK1*) and neuronal functioning (*MAPT*, *CLU*), providing further evidence that DNAm marks of childhood adversity are observable in adulthood.These findings have public health significance, as multiple studies of childhood adversity and epigenetic aging have shown that experiences of adversity accelerate DNAm aging (71, 80, 119, 136); however, more research is needed to determine how this relates to health outcomes and inequalities.

An often-cited limitation of epigenetic research using observational studies is its inability to make causal interpretations. Kandaswamy et al. (60) aimed to address this research gap in their study examining the association between childhood and adolescent victimization and longitudinal patterns of DNAm among monozygotic twins. However, no significant CpG sites were identified in paired analyses of monozygotic twins with discordant victimization experiences. The study included only a small sample of twins with discordant victimization experiences, which may have contributed to its ability to detect differences.

Overall, research on early life adversity suggests that exposures during prenatal and childhood periods may become biologically embodied through stress-related epigenetic pathways and may be observable into adulthood. Evidence also suggests that strong social support and networks may reduce the effects of childhood adversity on DNAm, though additional studies are warranted.

### 3.2. Adult Studies

A smaller body of research exists of psychosocial stressors in adulthood and DNAm. Here, we highlight two studies among Black women. In a small study of mother-child pairs, Black mothers experiencing high parenting stress had significant modifications at 95 CpG sites, including the *PARP-I* gene, which plays a role in response to stress (137). A separate study among the same study sample identified CpG sites at blood pressure-related genes in relation to stress and coping mechanisms; however, the results did not remain significant after accounting for multiple testing (23). Unfortunately, both studies were limited in their power to detect associations due to small sample sizes, warranting more research in larger samples.

## 4. DISCRIMINATION, RACISM, AND DNA METHYLATION

Discrimination and racism are recognized as important factors contributing to health inequalities (52, 134, 135). Although several articles propose frameworks for which experiences of discrimination and racism may shape epigenetic mechanisms (21, 49, 68, 69), we identified only four empirical studies of this topic (summarized in Table 1).

### 4.1. Childhood Exposure

Brody et al. (22) examined the effect of perceived racism during adolescence on epigenetic aging in young adulthood using data from two longitudinal cohort studies of Black families in rural Georgia. The authors found evidence that individuals exposed to racial discrimination had accelerated epigenetic aging. However, this association was modified by high support in the family environment. Although the durability of this effect is uncertain, these findings suggest that perceived racism during adolescence may accelerate biological aging, and this potentially harmful effect may be reduced or even prevented by a highly supportive family environment.

### 4.2. Adult Exposure to Racism and Discrimination

Three studies have examined associations between exposure to discrimination and racism in adulthood and DNAm patterns (14, 110, 125). Experiences of discrimination were associated with differential methylation at stress-related (i.e., *NR3C1*, *BDNF, FKBP5*) and inflammation-related (*LRRN3*) genes among Black and Latinx populations (14, 110, 125). These results suggest that interpersonal experiences of racism and discrimination may modulate DNAm at specific loci linked to stress pathways and associated with various health outcomes into adulthood.

## 5. SOCIOECONOMIC POSITION AND DNA METHYLATION

Social gradients in health are well recognized in the literature. Attention has recently shifted to better understand the mechanisms by which SEP leads to health inequalities. An extensive body of literature investigates whether measures of SEP (i.e., education level, household income, and occupational status) during childhood, adulthood, and across the life course influence DNAm. Here, we focus on some key studies (summarized in Table 1).

### 5.1. Early Life Socioeconomic Position

The impact of SEP during gestation,infancy,and childhood on DNAm patterns has received considerable attention (2, 3, 6, 19, 28, 42, 57, 70, 85, 95, 96, 109, 118, 121). Results from candidate gene analyses have identified differential DNAm in several stress-response pathways. Two studies identified links between maternal education and DNAm in CpG sites at the *HSD11B2* gene in both the placenta and peripheral blood in adults (3, 57). While DNAm was measured at different life stages, in utero and adulthood, the consistent findings support an epigenetic response to early life adverse social environments within *HSD11B2,* a gene responsible for stress response and cortisol inactivation. Moreover, Needham et al. (95) found that lower childhood SEP was associated with DNAm at three stress-related (*AVP*, *FKBP5*, *OXTR*) and two inflammation-related (*CCL1*, *CD1D*) genes. *AVP* and *FKBP5* encode proteins involved in the stress-response system and HPA axis functioning. DNAm in stress- and inflammation-related genes in response to adverse early life social environments is a plausible biological mechanism of health inequalities. In fact, Huang et al. (57) found that DNAm in *HSD11B2* was associated with body weight, total cholesterol, low-density lipoprotein cholesterol, and having a low birth weight offspring, all outcomes associated with known racial/ethnic differences.

The persistence of SEP-associated DNAm patterns found at birth into childhood has also been of interest in attempts to better understand the stability of socially driven epigenetic mechanisms across the life course. A large epigenome-wide association study (EWAS) using data from the Avon Longitudinal Study of Parents and Children (ALSPAC) mother-child cohort examined multiple socioeconomic variables, including maternal and paternal education and occupation status, in relation to DNAm at three time points: birth, childhood, and adolescence (2). Only maternal education was associated with differential DNAm, with four CpG sites mapping to three genes (*SULF1*, *GLB1L2*, *RPUSD1*) at birth and 20 CpG sites during adolescence. Although none of the CpG sites associated with maternal education overlapped across the life stages, two different maternal education–related CpG sites mapped to the *SULF1* gene at birth and in adolescence. This has biological relevance, as researchers have linked differential methylation within the *SULF1* gene to essential hypertension in young African American males (131). Similarly, Laubach et al. (70) examined the persistence of DNAm across birth, early childhood, and middle childhood in relation to prenatal SEP (measured as an index of maternal education, marital status, income, receipt of public assistance, neighborhood income, and percent below poverty level), and they found that 29 CpG sites at birth were associated with low prenatal SEP. Of these, only one remained significant in early childhood, *LRRN4*, and none in middle childhood. *LRRN4* expression has been linked to schizophrenia and heart disease (76, 129), which disproportionately burden individuals of lower SEP (33, 89, 92).

Evidence from an EWAS of prenatal SEP and DNAm suggests associations may be sex-specific (109). In a study of infants born before 28 weeks of gestation, 27 significant CpG sites in placentas from female pregnancies were found in response to prenatal SEP, but only 2 CpG sites were found in placentas from male pregnancies. Moreover, Appleton et al. (3) found more associations between SEP and methylation at the *HSD11B2* gene in male placentas than in female placentas. These findings further demonstrate the need for additional research on sex-specific epigenetic pathways.

While decades of research demonstrate the long-term consequences of low childhood social position, recent studies suggest that the effects on epigenetic aging continue well into adulthood, but findings are mixed. Two separate studies found that low parental occupational status during childhood was associated with accelerated epigenetic aging in adulthood (6, 47). These findings differ from those of a more recent study observing no association between parental occupational status during childhood and epigenetic aging in adulthood among participants in the Irish Longitudinal Study on Aging (85). Several factors may explain these contrasting findings, including differing social contexts, which we discuss below (see the section titled Future Research).

### 5.2 Adult Socioeconomic Position

Like early life SEP, low adult SEP is associated with differential DNAm levels at stress- and inflammation-related genes, including at the *AVP* gene (stress-related pathway) and the *CD1D, F8, KLRG1, NLRP12,* and *TLR3* genes (inflammation-related pathways) (95). Furthermore, studies of SEP and epigenetic aging in adulthood demonstrate effects across epigenetic clocks, with low SEP attainment associated with accelerated DNAm aging across several epigenetic clocks, including Hannum’s clock,Levine’s PhenoAge,GrimAge,and (with weaker evidence of associations) Horvath’s clock (30, 44, 47, 115).

### 5.3. Life Course Socioeconomic Position

Evidence exists that one’s SEP trajectory across the life course (from childhood to adulthood) influences DNAm in adulthood. This has significant implications for health inequalities given that racially minoritized groups are less likely to achieve upward social mobility in the United States (112). Needham et al. (95) found associations between persistent low SEP (across childhood and adulthood) and differential DNAm in stress-related (*AVP*, *FKBP5*, and *OXTR*) and inflammationrelated (*CCL1*, *CD1D*, *F8*, *KLRG1*, and *NLRP12*) genes. The authors also identified associations withgene expressionacrossseveral genes(95).Similarly,Stringhiniet al.(118)found linksbetween SEP trajectories and DNAm patterns in inflammation-related genes (*NFATC1*, *MAP3K6*, *IL1A*, *GPR132*, *CXCL2*, and *MAP2K5*). A more recent EWAS of life course SEP trajectories identified 2,546 statistically significant CpG sites associated with low SEP across childhood and adulthood (1,777 sites with increased methylation and 769 sites with decreased methylation), 1 CpG site associated with upward mobility, and no CpG sites associated with downward mobility (86). Collectively, these studies underscore two critical insights: Childhood is a sensitive period of the life course, during which exposure to low SEP can have long-term effects on epigenetic mechanisms; and social mobility has limited potential impact on the epigenome.In fact,George et al.(47) found that adults experiencing lower SEP in childhood had accelerated epigenetic aging, regardless of their SEP in adulthood.

## 6. NEIGHBORHOOD SOCIAL ENVIRONMENT AND DNA METHYLATION

Recent evidence suggests that health inequalities produced by adverse neighborhood contexts are also epigenetically mediated (51, 107). Below, we highlight studies of neighborhood social environmental characteristics and DNAm patterns and epigenetic aging (summarized in Table 1).

### 6.1. Early Life Neighborhood Social Environment

Research on the effects of neighborhood-level social exposures on the epigenome in utero and during early childhood is limited. Studies of newborns living in disadvantaged neighborhoods found higher global methylation; higher cord blood leukocyte DNAm of a cancer-relevant gene, *MEG3*; and differential DNAm at the *SLC6A4* gene (28, 34, 63). Although these studies were unable to evaluate associations between census tract disadvantage and later-life disease phenotypes, they offer realistic biologic pathways between elevated gestational stress, epigenetic modifications, and later-life risk of cancer (e.g., *MEG3* expression) and poor mental health (e.g., *SLC6A4* expression) (34, 63).

A different body of work has evaluated the effect of childhood and adolescent neighborhood exposures on the adult epigenome. McDade et al. (87) evaluated the effect of infant and childhood social and ecological exposures on DNAm at 114 target inflammation-related genes in Cebu, Philippines, and they found significant methylation differences in CpG sites at *C1S*,*GNG2*, *CD8A*, *APBA2*, *EGR4*, *TLR1*, *IL-1A*, *PIK3C2B*, and *SULT1C2*. Of note, they collected inflammatory biomarkers concurrently and found that lower DNAm at *C1S* and *PIK3C2B* and higher DNAm at *TLR1* were associated with generally increased inflammatory markers.The inclusion of both epigenetic and biomarker data is a significant strength and suggests that neighborhood-level environmental adversity may contribute to epigenetic modifications that result in phenotypic differences in the adult inflammatory response. Reuben et al. (104) conducted a similar analysis in 18 candidate stress- and inflammation-related genes, finding only one site at *NLRP12* that was significantly associated with neighborhood disadvantage. An additional analysis using the same data found that neighborhood disadvantage predicted higher epigenome-wide scores related to smoking and inflammation (104). Additionally, studies of neighborhood social and economic variables have also examined DNAm age estimators, finding accelerated DNAm aging among those exposed to childhood neighborhood disadvantage (58, 80).

### 6.2. Adult Neighborhood Social Environment

There is a relatively small body of research regarding the effect of neighborhood exposures on epigenetic mechanisms in adulthood. Smith et al. (116) evaluated the effect of neighborhood socioeconomic disadvantage and social cohesion on DNAm of seven stress-related genes and 11 inflammation-related genes. They found that neighborhood socioeconomic disadvantage was associated with DNAm at three stress-related (*AVP, CRF*, and *SCL6A4*) and three inflammationrelated (*F8, LTA4H*, and *TLR1*) genes, whereas neighborhood social cohesion was associated with DNAm at four stress-related (*AVP, BDNF, FKBP5*, and *SLC6A4*) and seven inflammation-related (*CCL1, CD1D, F8, KLRG1, NLRP12, SLAMF7*, and *TLR1*) genes. It is notable that Reuben et al. (104) used the same gene set in their analysis of 18-year-olds in the United Kingdom and found an association with neighborhood disadvantage only at *NLRP12*.

Rather than adopting a candidate gene approach, however, the majority of the literature investigates the effect of neighborhood stressors on epigenetic aging. Multiple studies observed accelerated DNAm aging in relation to adverse neighborhood social environment (72, 74, 81, 132). Two separate studies found that positive aspects of the social environment modified the effects of neighborhood stressors on accelerated DNAm aging. Martin et al. (81) found associations between poor neighborhood quality and accelerated DNAm age; however, associations were only observed among participants living in neighborhoods with low social cohesion. WardCaviness et al. (132) found that poor neighborhood quality was associated with an increased epigenetic mortality risk score (eMRS). However, stratification on the presence of large, mature trees (an indicator of greenspace) in the neighborhoods strongly attenuated the effects of the poor neighborhood environment, such that participants living in neighborhoods with large, mature trees were indistinguishable from the referent group living in superior neighborhood environments. These findings add to the previously described evidence that positive aspects of the social environment may offset the effects of adverse social exposures on DNAm patterns.

## 7. EPIGENETIC MECHANISMS: A MECHANISTIC LINK TO HEALTH INEQUALITIES

So far, this review has focused primarily on research linking social experiences to DNAm; but how does this relate to health inequalities? A small body of literature exists on racial differences in DNAm patterns and health outcomes. In this section, we highlight several of these studies. Salihu et al. (108) examined whether candidate CpG sites associated with preterm birth were differentially methylated among infants of Black mothers compared to non-Black mothers, finding that three CpG sites at the *TNFAIP8* and *PON1* genes significantly differed between the two groups. Wang et al. (130) examined methylation levels in CpG islands of candidate genes among Black and White breast cancer patients and found racial differences in methylation for the *CDH13* gene, which were more pronounced among Black patients with early onset of estrogen receptor (ER)-negative breastcancer comparedtomatched Whitepatients.Moreover,methylation levelsat three genes (*CDH13*,*RASSF1A*,and *RARb2*) were higher among Black women than among White women and were associated with lower survival (130). Devaney et al. (37) examined genome-wide DNAm differences in prostate cancer tissue versus normal prostate tissue and found more differentially methylated CpG sites in African American men (2,973 CpG sites) than in Caucasian men (745 CpG sites) and a smaller number of overlapping sites across the two groups (330 CpG sites).Differential DNAm patterns related to metabolic syndrome have also been found in African American and White individuals,with identified CpG sites linked to breast and colon cancers (27). Of the differential DNAm sites identified among African Americans, one CpG site at the *ABCG1* gene was also previously found in a separate study of differential DNAm and metabolic syndrome among African American adults (1). Lastly, epigenetic aging was assessed as a potential mediating factor of inequalities in mortality by race/ethnicity using data from the Women’s Health Initiative (77). Non-Hispanic Black women had higher risk of mortality compared to non-Hispanic White women; yet, this association was partially attenuated once differences in DNAm age were accounted for. Elucidating differences in DNAm patterns and health outcomes across racially minoritized groups can lend critical insights into the epigenetic pathways of health inequalities; however, such research does not explicitly address the social and structural origins of those differences.

To date, few empirical studies exist testing whether social exposure–related DNAm changes relate to health inequalities in the United States. Vidal et al. (127) explored the role of DNAm at imprint regulatory regions in associations between prenatal stress and preterm birth. Although maternal stress was not associated with preterm birth in their study, they found that higher prenatal stress was associated with increased offspring methylation in the *MEST* DMR, and the associations differed for male and female offspring. Unfortunately, the authors did not examine this association by race/ethnicity. Straughen et al. (117) examined whether *IFG1* methylation mediated associations between maternal race and birth weight. In their study, they found that Black women had babies born at significantly lower weights and with higher *IGF1* methylation compared to non-Black mothers. Mediation analyses found that *IGF1* partially mediated the association between maternal race and birth weight. Results from these two studies are promising; however, more research is needed that explicitly examines social exposures produced by inequitable and racialized systems in relation to DNAm and health inequalities to truly begin to understand the epigenetic mechanisms of health inequalities.

## 8. FUTURE RESEARCH

In this review, we highlighted how DNAm is shaped by social experiences and discussed studies of racial differences in DNAm patterns and health outcomes. Below, we offer suggestions for future social epigenetics research.

### 8.1. Race and Ethnicity in Epigenetic Research

Researchers have long suspected epigenetic pathways act as a mechanism of health inequalities (7, 49, 69, 97, 98, 107, 126); our recommendations for future research on social epigenetics and health inequalities aim at elucidating more clearly these epigenetic pathways and strengthening this literature. First, many studies included in this review either excluded non-White participants or made no mention of race or ethnicity,and they comprised samples outside of the United States. Researchers need to extract information from the commonalities in social epigenetics about both societal implementation of policies that lead to health inequalities and the biological mechanisms that mediate such effects. Although the social constructs of race and ethnicity are often time- and location-dependent, when utilized as social determinants of health, they are proxies for systemic racism and discrimination, which occur globally. As much as the apartheid system of South Africa may have differed from the Jim Crow era of the United States,there are commonalities to be found in the health inequities introduced by these (and many other) systems built on racism, discrimination, and oppression. Because epigenetic mechanisms are highly evolutionarily conserved, we may still extract useful information on causal mechanisms linking experiences of structural racism and discrimination to epigenetically mediated health inequalities from disparate global audiences. However, extracting such information from source populations and extrapolating to structural racism and discrimination occurring in other contexts require causal study designs with carefully considered and locale-specific proxies backed by mechanistic insights, which are often lacking. Thus, we must continue to be highly cautious in generalizing results or assuming that the underlying biological mechanisms proposed by a study in one population will translate to others. In addition, it is absolutely critical to develop a racially and ethnically diverse literature of social epigenetics backed by causal study designs—where possible—to better understand how epigenetics influence health inequalities throughout the life course.

Second, because social epigenetics research brings together researchers from multiple disciplines, foundations in concepts of race and ethnicity are important. Race is a social, not biological, construct used to assign people into a social hierarchy based on physical or imagined features (35, 65, 106). Like race, ethnicity is a social categorization of people with similar beliefs, culture, language, and religion (99). Race and ethnicity have correlations with genetic ancestry due to geographic origins and, therefore, may capture information about genetic variation (12). Although epigenetics does not follow the same inheritance patterns as genetic variation, it is still influenced by genetic variation and in some cases may be passed down via epigenetic imprinting during embryonic development. Importantly, unlike genetic variation, aspects of ethnicity, such as shared cultural experiences, may be passed down through epigenetic mechanisms, making epigenetics correlated with both genetic and cultural ancestry. Although these linkages are complex and may be difficult to disentangle within any individual study,it is imperative that the field of social epigenetics acknowledge and address them wherever possible. Clearly defined hypotheses, targeted measures (e.g., genotyping and assessments of experienced racism), and a willingness to clearly delineate the limitations of a study will help clarify a study’s scope and results.Additionally, researchers from all fields must recognize that social constructs are strong drivers of health inequalities. Any study proposing a biological basis for health inequalities with known social drivers must be held to a high standard and backed by strong data (e.g., genotyping and decomposition of genetic ancestry and race/ethnicity) due to the ongoing history of abuse and misuse of such claims.

Lastly, the field of social epigenetics seeks to determine epigenetic changes occurring among groups as a result of adverse social experiences and inequities that produce population health inequalities. As such, studies should carefully consider context and history when analyzing associations by race and ethnicity, and they should specifically discuss the concepts of racism and discrimination that link race and ethnicity to ground the studies in causal social mechanisms. Studies of race and ethnicity must understand how historical and contextual factors (i.e.,country of origin, historical exposures,colonization,oppression,and immigrant or mainland discrimination) are heterogenous, and any categorization into broad groups with assumed shared experiences may miss highly salient factors that affect both health inequalities and epigenetics. Thus, a strong consideration and understanding of the histories of racial and ethnic groups are needed to contextualize the meaning of methylation differences in social epigenetics research.

### 8.2. Understanding Sex-Specific Associations

Another recommendation for future studies is to investigate sex-specific DNAm responses to adverse early life social environments. Health inequalities based on biological sex (as well as gender identity) often intersect with (and possibly amplify) race- and ethnicity-based health inequalities. Biological sex differences in stress response and vulnerability to stress across early and late life periods have led to differences in short- and long-term health outcomes (11,120).Sex-specific stress response can be attributed to both circulating gonadal hormones and genetic sex (10). The placenta plays an integral role in sex differences in early life programming of stress-response pathways (10, 40). Whereas sex in early life is recognized as a biological variable, gender identity may also give rise to health inequalities relevant to social epigenetics. Thus far, adverse social experiences related to gender identity have not been studied in the context of epigenetics. Future research should incorporate perspectives of intersectionality (20) to better understand the broad implications of social identities and experiences on epigenetic mechanisms and health inequalities across the life course.

### 8.3. Enhancing Social Epigenetic Study Designs

Study design improvements form the center of our final set of recommendations for future social epigenetics research. As the field of social epigenetics rapidly emerged, studies were primarily of associations at a single point in time, likely due to the paucity of epigenetic data, particularly across multiple time points. Given the responsiveness of DNAm to environmental exposures, it is reasonable to believe methylation patterns will change as social exposures change over the life course. Only a handful of studies described in this review investigated changes in social experiences and DNAm over time (2, 39, 60, 70, 80, 86, 95, 118); however, as research continues to expand, prospective cohort studies with methylation data at multiple time points are needed to make interpretations of temporality and causality. Additionally, more research is needed to understand the functional relevance of differential methylation patterns in response to social exposures. Many researchers have incorporated measures of gene transcription and health outcomes into their studies, which helps to determine whether identified methylation patterns manifest as changes in gene expression—the primary biological mechanism through which methylation acts. This type of cross-omics validation is key to building evidence that can lead to social epigenetics having clinical, policy, or therapeutic impacts. Lastly, as prospective, longitudinal studies with social exposures, DNAm, gene expression, and health outcomes become available, improved statistical methods are needed to fully address the goal of understanding how social experiences and exposures affect DNAm patterns and influence health inequalities across the life course.

## 9. CONCLUSION

There is growing interest in epigenetics research among social scientists because of its proven sensitivity to exogenous exposures and its role in regulating gene expression. Mounting evidence exists demonstrating that adverse social exposures, such as maltreatment, crime, racism, discrimination, and neighborhood poverty, influence DNAm patterns, particularly during early life periods (i.e., gestation, infancy, and childhood) when the brain and other biological systems are still developing. The evidence that socially induced DNAm changes are related to biological mechanisms like the immune and stress (e.g., glucocorticoid) pathways during periods of high vulnerability has important implications for long-term health trajectories and inequalities, warranting more longitudinal investigations with information across early and later life stages.This review underscores the need for additional research in racially and ethnically diverse cohorts to determine whether DNAm patterns shaped by adverse social environments and exposures result in disparate population health trajectories and disease patterns. Modifications in DNAm patterns with respect to adverse social exposures across the life course are also observable in adulthood. Chronic diseases typically manifest in young to middle adulthood, with earlier onset among racially minoritized groups. Whereas existing studies have identified racial differences in DNAm patterns, which may contribute to these health outcomes, additional considerations of social and economic experiences of race and ethnicity are needed to elucidate the epigenetic mechanisms of health inequalities. Findings from social epigenetics research have the potential not only to identify molecular drivers and mechanisms of health inequalities, but also to represent a rapid marker of when structural interventions are having the desired biological/health effect on communities. DNAm or other epigenetic biomarkers may serve as early indicators of reductions in adverse health outcomes or mortality; thus, they can inform researchers and policy makers about effective (or ineffective) interventions for improving health inequalities.

## Figures and Tables

**Table 1:** Summary of findings from social epigenetic studies

Exposure	Exposure operationalization	Candidate genes^a^	Epigenetic aging	References^b^
Maternal/prenatal adversity	Prenatal exposure to maternal psychosocial stressors including daily stressors, cumulative lifetime stress, financial stress, war-related stress, adverse childhood events, significant life events, abuse, and exposure to violence	*ADAM10, AP2A2, BARX1, BDNF, CFTR, CORIN, CRH, CRHBP, FBXO30, FKBPF, IGF1, MEST, NR3C1, NUDT16P, PRDM2, SDHAF2, SMYD3, STON1*	NA	24, 61, 62, 90, 91, 100, 101, 103, 111, 127, 138
Child adversity	Adverse childhood events including physical, emotional, or sexual abuse; neglect; parental absence; parental illness or disability; suboptimal maternal bonding; parental death in childhood; and childhood physical illness	*5HTT, ASPSCR1, BRD7, C11orf49, C15orf26, C19orf30, C5orf21, C5orf66, C8orf31, CCNF, CLU, CPA6, CRMP1, DENND1C, DNAAF5, GNAQ, GPATCH2, GPR61, HERPUD1, HP1BP3, KIF26A, LINC01182, LOC101929555, MAPT, METAP1, MGC42630, MGC4562, MGMT, NEDD9, NPY, NRC31, NT5C1B, OR2G3, OXTR, PCDH15, PHACTR2, PKN1, PLBD1, PRDM16, PRR14, RASA2, RASGRF2, RSPH14, SFRP1, SLC6A4, SYCE1, THSD4, TK1, TM6SF2, TMEM156, TMEM67, TONSL, TRDN, VPS28, WNT6*	Child adversity associated with accelerated epigenetic aging	16, 17, 39, 41, 55, 60, 64, 71, 80, 84, 105, 114, 119, 124, 136
Adult psychosocial stress	Stress overload or parenting stress	*BACH2, CCDC90B, CHADL, EPAS1, GJA10, GJB3, KY, MGLL, MIR1273H, TNR, VANGL2, WDR19*	NA	23, 136, 137
Socioeconomic position	Education level, household income, assets, or employment status	*AC006033.2-4, AC069360.7, AC074130.3, AC091817.6-1, AC099849.4, AL163195.5, AL391427.9-2, AP000753.4, AVP, C15orf26, C18orf63, CCL1, CD1D, CD44, CDH4, CHST15, CXCL2, DLGAP2, DR1, EZH2, F8, FJX1, FKBP5, GPR132, GRAMD4, HSD11B2, IL1A, KLRG1, MAD1L1, MAP2K5, MAP3K6, MEFV, NFATC1, NLRC5, NLRP12, OXTR, PPP2R2D, SFRS8, SPARC, TLR3, TMEM158, UBE4A, ZNF827*	Low childhood socioeconomic position associated with accelerated epigenetic aging; low adult socioeconomic position associated with accelerated epigenetic aging	2, 3, 6, 19, 28, 30, 44, 47, 57, 70, 85, 86, 88, 95, 96, 109, 115, 118, 121
Racism/ discrimination	Perceived exposure to interpersonal racism or discrimination	*ALOX15P1, ANKRD63, ARHGAP15, BDNF, CYFIP1, FAT2, FKBP5, hCG_2003567, IMMP2L, LOC101928443, LRRN3, MADILI, NR3C1, SORCS1, STF2D3, WWOX, ZXDC*	Perceived discrimination associated with accelerated epigenetic aging	14, 22, 110, 125
Neighborhood social environment	Living in an environment characterized by concentrated disadvantage and poverty, violence and crime, or disorder	*5-HTT, AHRR, APBA2, AVP, BDNF, C1S, CCL1, CD1D, CD8A, CNTNAP2, CRF, CYP1A1, EGR4, F8, FKBP5, GNG2, IL-1A, KLRG1, LTA4H, MEG3, NLRP12, OR4C13, PIK3C2B, SLAMF7, SLC6A4, SULT1C2, TLR1*	Neighborhood disadvantage associated with accelerated epigenetic aging	28, 34, 58, 63, 72–74, 80, 81, 87, 104, 116, 132

Abbreviation: NA, not available.

a The list of candidate genes is compiled from candidate gene studies and genome-wide studies (only select candidate genes are shown). The list provided here is not exhaustive; please refer to the specific studies for full lists.

b The references listed also include relevant studies that are not explicitly described in this review.
